# Carcass Quality Profiles and Associated Genomic Regions of South African Goat Populations Investigated Using Goat SNP50K Genotypes

**DOI:** 10.3390/ani12030364

**Published:** 2022-02-02

**Authors:** Keabetswe Tebogo Ncube, Edgar Farai Dzomba, Khanyisile Hadebe, Pranisha Soma, Lorinda Frylinck, Farai Catherine Muchadeyi

**Affiliations:** 1 Agricultural Research Council, Biotechnology Platform, Private Bag X5, Onderstepoort, Pretoria 0110, South Africa; keabetswe.ncube@gmail.com (K.T.N.); MdladlaK@arc.agric.za (K.H.); 2 Discipline of Genetics, School of Life Sciences, University of KwaZulu-Natal, Private Bag X01, Scottsville, Pietermaritzburg 3209, South Africa; Dzomba@ukzn.ac.za; 3 Agricultural Research Council, Animal Production, Private Bag X2, Irene 0062, South Africa; Pranisha@arc.agric.za (P.S.); Lorinda@arc.agric.za (L.F.)

**Keywords:** candidate genes, carcass quality, goats, goat ecotypes, GWAS, SNP50K

## Abstract

**Simple Summary:**

South Africa is one of the major goat producing countries on the African continent. However, despite a large number of goats being produced, there is still a growing demand for chevon, which leads to producers being unable to reach demand, resulting in an absence of chevon in retail markets. Carcass quality is an important economic trait that plays a major role in influencing consumer preferences and high nutrient provision. Even though chevon is an easily accessible meat for smallholder farmers and has health benefits, it is still less preferred due to perceptions of low meat quality attributes such as toughness, off-odours and flavour, and unappealing colour. The majority of goat populations are village ecotypes whose genetic potential for meat and carcass quality is unknown.

**Abstract:**

Carcass quality includes a battery of essential economic meat traits that play a significant role in influencing farmer breed preferences. A preliminary study was undertaken to investigate the carcass quality and the associated genomic regions in a small nucleus of animals that are representative of South African goat genetic resources. Samples of the South African Boer (*n =* 14), Northern Cape Speckled (*n =* 14), Eastern Cape Xhosa Lob ear (*n =* 12), Nguni/Mbuzi (*n =* 13), and Village (*n =* 20) were genotyped using the Illumina goat SNP50K and were phenotyped for carcass quality traits. SA Boer goats had heavier warm and cold carcass weights (17.2 ± 2.3 kg and 16.3 ± 2.3 kg). Pella village goats raised under an intensive system had significantly (*p* < 0.05) heavier warm and cold carcass weights (9.9 ± 1.1 kg and 9.2 ± 1.2 kg) compared to the village goats that are raised extensively (9.1 ± 2.0 kg and 8.4 ± 1.9). A total of 40 SNPs located on chromosomes 6, 10, 12, 13, 19, and 21 were significantly associated with carcass traits at (−log10 [*p* < 0.05]). Candidate genes that were associated with carcass characteristics (*GADD45G*, *IGF2R*, *GAS1*, *VAV3*, *CAPN8*, *CAPN7*, *CAPN2*, *GHSR*, *COLQ*, *MRAS*, and *POU1F1*) were also observed. Results from this study will inform larger future studies that will ultimately find use in breed improvement programs.

## 1. Introduction

The world goat population is approximately 1 billion [1], with the South African industry producing about 7.8 million goats [2,3]. South African goat production contributes about 3% of the goats in Africa as a whole [4]. About 63% of the goats in South Africa are non-descript and unselected populations that are raised in communal farms in rural areas [3]. There is a high demand for goat meat on the African continent; however, this demand is unmatched by existing goat populations. Reasons for this disparity include low growth potential and carcass quality that is affected by genetic and environmental factors [5]. South Africa is one of the few countries that has developed meat-type breeds from their own indigenous genetic resource that have been recognized as commercial breeds. These include the South African Boer, Savanna, and Kalahari Red goats [4,6,7]. Breeds such as the Boer dominate the commercial industry and have good carcass quality and high carcass yields of 25 kg for bucks and 22 kg for does 100 days post-natal [8]. Morrison [9] described the Northern Cape Speckled, Mbuzi/Nguni, and the Eastern Cape Xhosa Lob ear as the indigenous veld goat populations that are kept by the Indigenous Veld Goats (IVG) association farmers in organized farming and breeding societies that are usually under semi-intensive production systems. Additionally, there are uncharacterized village ecotypes that are raised by communal farmers that have not been developed for any traits and that have multipurpose functions, i.e., for their meat, milk, and skin [7,10]. The carcass quality characteristics of Indigenous Veld Goats (IVG) and village goats have not been reported. Despite being a major goat producer, the country contributes <1% of chevon production in the South African red meat market [11]. This low production may be because chevon is regarded as inferior meat through market preferences/perceptions and because of a shortage of product supply [1].

The primary role of goats in African countries is meat (chevon), which serves as a source of easily accessible protein for poor communities [10]. Although chevon has excellent meat quality characteristics and health benefits, it is the least preferred among consumers, which is mainly due to unfavourable perceptions that are related to toughness and strong odour [12]. With the majority of goats being kept by communal farmers in rural areas, poor growth performance, which subsequently leads to low carcass yields, remains a challenge in the chevon industry [13]. Low chevon production is a growing concern in South Africa. Some of the causes of this low productivity are because the majority of the indigenous goats in Africa are farmed in rural areas that are characterized by inefficient feeding and management, disease constraints, a lack of characterization, and the inadequate exploitation of genetic resources [5]. Limited information exists on the genetic factors/potential that influence the increasing productivity in indigenous goats raised in communal areas. The differences in the growth performance and carcass characteristics of different breeds under similar or varying production systems is poorly understood. Village goat ecotypes have not been adequately characterised in terms of their growth and carcass quality attributes. There have been limited efforts made for the commercial promotion of chevon as well as the development of uncharacterized goat populations for chevon production. There are no reported/written records that describe the carcass characteristics and genetics of carcass quality traits of indigenous goats, including South African populations.

Several goat genomic tools can be used to unravel the genetic potential of breeds and populations. High-throughput targeted gene sequencing of the growth hormone 1 gene has shown the locus to be polymorphic and capable of differentiating goat populations from different breeds and production systems [10]. The Illumina goat SNP50K is a medium-density SNP chip consisting of markers that are evenly spaced across the goat genome and is a useful tool for population genetics and association studies [7,14,15]. The Illumina goat SNP50K Bead chip has been used for body morphological traits in Sudanese goats [16]. Using this chip, the SNPs and genes that are associated with growth, body metabolism, and other adaptive traits were reported to be segregating and differentiating goat populations from different geographic and production environments of South Africa [17]. This pilot study aimed to investigate the genetics of carcass traits in five South African indigenous goat populations and was inclusive of the uncharacterized village ecotypes from Pella village. This was achieved by first investigating the association between the breed and carcass quality traits followed by a genomic association analysis to investigate the genes and molecular mechanisms associated with carcass quality in different breeds and populations using the goat SNP50K genotypes.

## 2. Materials and Methods

### 2.1. Animal Description and Management

The Animal Ethics Committee of Agricultural Research Council, Animal Production, South Africa, has approved all of the work and animal management undertaken in this study (Ethics approval number “APIEC16/010”).

The study involved four well-established goat breeds: the South African Boer (SAB) (*n =* 14); Northern Cape Speckled (NCS) (*n =* 14); Eastern Cape Xhosa lob ear (XL) (*n =* 14); and Nguni/Mbuzi (MBZ) (*n =* 13), which were purchased and sampled from farmer members of the Indigenous Veld Goat Society (IVG) at 12 weeks of age and were brought to the experimental farm at the Agricultural Research Council, Animal Production (ARC-AP), South Africa. Goat kids were weighed on arrival and had weaning weights ranging from 5 kg to 15 kg. These goats were kept in a browsing camp at the Small Stocks Unit of the ARC-AP. They were kept on a management diet of game pellets (110 g/kg, 25–70 g/kg crude fat, 110–200 g/kg crude fiber, 6–10 g/kg calcium, 2.5 g/kg phosphorus and 3.68% non-protein nitrogen) provided at 3% of live weight/animal/day. Lucerne hay and clean water were available ad libitum. They were evaluated for signs of diseases, and in cases of diseases, treatment was either administered, or the animal was taken to a veterinarian or hospital. Dipping with acaricides was performed twice a month to prevent external parasites. 

In addition, village goat ecotypes (*n =* 28) were purchased and sampled from Pella village, which is located in the North West province of South Africa, at a weaning age of 12 to 12.5 weeks. The animals were sampled from 14 farms, and 2 kids of similar age were purchased per farm. A proportion (*n =* 14) of these village goats (VTI) were moved to the Small Stocks Unit of ARC-AP, where they were weighed on arrival and were raised similarly to the other breeds of IVG described above. The second set of these goats (VTE; *n =* 14) was raised under typical communal farming systems at their respective village farms. They were penned at night and left to forage during the day. There was no feed supplementation given to this set of animals. Due to considerable variation in breeds and management practices between farms coupled by the small goat flock sizes/farm, only a few animals from farmers with similar production and management systems could be sampled and utilized in this analysis.

### 2.2. Blood Collection, DNA Isolation and SNP Genotyping

Venous blood (2 mL) was collected at 24 weeks of age from the jugular vein of 72 goat kids out of the 83 animals present at the start of the experiment, and these samples were transported in an icebox to the Agricultural Research Council, Biotechnology Platform laboratories and were stored at −20 °C in a freezer until further use. These 72 animals consisted of SAB (*n =* 13), NCS (*n =* 14), XL (*n =* 13), MBZ (*n =* 12), (VTI *n =* 9), and VTI (*n =* 11).

DNA was isolated using the optimized Qiagen DNeasy blood and tissue kit (www.qiagen.com (accessed on 19 July 2017)) according to manufacturer’s instruction with modifications such as increasing the sample volume to 200 µL blood and an increased incubation period of 120 min. DNA quantification was performed on the Qubit^®^ 2.0 Fluorometer using the Invitrogen’s Qubit^TM^ dsDNA BR Assay Kit (Invitrogen, Life Technologies, Carlsbad, CA, USA). The quality of the DNA was investigated electrophoretically on 1% agarose gel and with 4 µL ethidium bromide at 80 V for 30 min. After isolation and quantification, 50 ng/µL genomic DNA of 72 goats were genotyped on the Illumina Goat SNP50K BeadChip using the Infinium assay. The SNP chip was scanned on the Illumina IScan genotyping platform, and the SNP genotypes were called using the genotyping module in GenomeStudio™ V2010.1 (Illumina Inc., San Diego, CA, USA).

The SNP marker map file was updated using the Golden Helix SNP Variation Suite version 8.7.2 (Golden Helix, Inc., Bozeman, MT, USA). PLINK v1.09 [18] was used to filter individual animals with genotypes of a 95% call rate. SNPs were pruned for call rate ≤ 95%, minor allele frequency (MAF ≤ 0.05), and deviation from Hardy–Weinberg equilibrium (HWE; *p* < 0.001). For population structure analysis, linkage disequilibrium (LD) pruning was performed at (LD > 0.2) as well as for related individuals (IBD > 0.5).

### 2.3. Slaughter Procedure and Carcass Quality Measurements

All of the kids were raised until they were 36 weeks old, then slaughtered using approved procedures at the ARC-AP abattoir. Kids that had been raised at the ARC-AP were transported from the Small Stocks unit to the holding pens at the ARC-AP Abattoir, where they were kept overnight with a 24 h pre-slaughter fasting period. Water was freely assessable. Extensively raised kids were transported from Pella village, North West, to the holding pens at the ARC-AP Meat Science Building abattoir, where they were kept overnight before slaughter, as described above. Pre-slaughter weight (WS) was measured 24 h before the animals were transported to the abattoir to be slaughtered.

Goats were electrically stunned for 5 s at 200 volts, rendering them unconscious, after which they were slaughtered, skinned, and allowed to bleed for 5 min by suspension by both Achilles heels [19]. After bleeding, the head was cut at the neck point from the spinal column at the occipital-atlantal joint followed by the removal of the trotters at the joint from the metacarpus and the ulna of the forelimbs and the joint between the metatarsus and the fibula in the hind limbs. The offal were removed from the abdominal cavity during evisceration and were not included in this study. Seven to eight goats were slaughtered per day, and all of the animals were slaughtered at the same abattoir at ARC-AP under the same prescribed conditions within a 1-week period. 

Following evisceration, the head, trotters, and offal were removed from the carcass. Warm carcass weight (WCW) was measured one-hour postmortem before the carcass was chilled by hanging it from both hind legs, and the carcass was immediately chilled at 4 °C. Cold carcass weight (CCW) was measured as the weight of chilled carcass 24 h postmortem. The kidneys, kidney fat, and tail were removed after the carcass was chilled and were not used in this study. The fat code classification and distribution of the subcutaneous fat were performed by a visual appraisal of the carcass by a trained official who then assigned the carcasses a fat code (FC) according to the South African classification of red meat guidelines (http://www.samic.co.za/downloads/Redmeat.pdf (accessed on 10 October 2017). The fat codes for the studied goats ranged from 0–2 and were in increments of 0.25.

Dressing percentage (DP) and chilling loss percentage (CL) were calculated as:

$$\mathrm{DP}\;(\%)=\frac{\mathrm{CCW}}{\mathrm{WS}}\;\times \;100$$

$$\mathrm{CL}\;(\%)=\frac{\mathrm{WCW}-\;\mathrm{CCW}}{\mathrm{WCW}}\;\times \;100$$

where CCW is the cold carcass weight, WS is the weight before slaughter after fasting for 24 h, and WCW is the warm carcass weight. 

### 2.4. Statistical Analysis of Carcass Quality Data

Least square means and standard errors of the carcass quality measurements were estimated using the general linear model procedure (PROC GLM) within the Statistical Analysis System (SAS Institute Inc., Cary, NC, USA). The model factored in breed and sex as well as their interaction. 

### 2.5. Association Analysis

For those traits that were significantly affected by breed, a mixed linear model (MLM) in Golden Helix SNP Variation Suite version 8.7.2 (Golden Helix, Inc., Bozeman, MT, USA) was used to investigate the SNPs that were significantly associated with various carcass traits using the model:

y=Xα+Kμ+e

where *y* was the carcass trait, *X* was the genotype (15,711 SNPs), *α* was regarded as the vector of the fixed effect (breed) while *K* was the relative kinship matrix, μ was regarded as the unknown random effect, and *e* was the random error. The Bonferroni correction threshold for multiple tests was used to detect the genome-wide significant SNPs, which were defined as *α*/*K* (*α* = 0.05 and *K* is the number of SNPs = 15,711).

The dressing percentage was excluded in the association analysis because there were no significant differences between breeds/populations.

### 2.6. Gene Annotation and SNP Association

The Ensemble BioMart tool (https://www.ensembl.org (accessed on 21 October 2017)) was used to identify genes within a 1 Mb region of the significant SNP (−log10 (*p* < 0.05)). All of the genes were searched against the Kyoto Encyclopedia of Genes and Genomes (KEGG) pathways.

## 3. Results

### 3.1. SNP Genotype Quality Control and Population Structure

After quality control, 69 individuals with 49,000 SNPs were retained for downstream analysis. For the population structure and admixture analysis, 47 individuals were retained after LD pruning and IBD for the removal of related individuals and outliers. For the association analysis, 15,711 SNPs and 53 animals were used.

**Figure 1:**
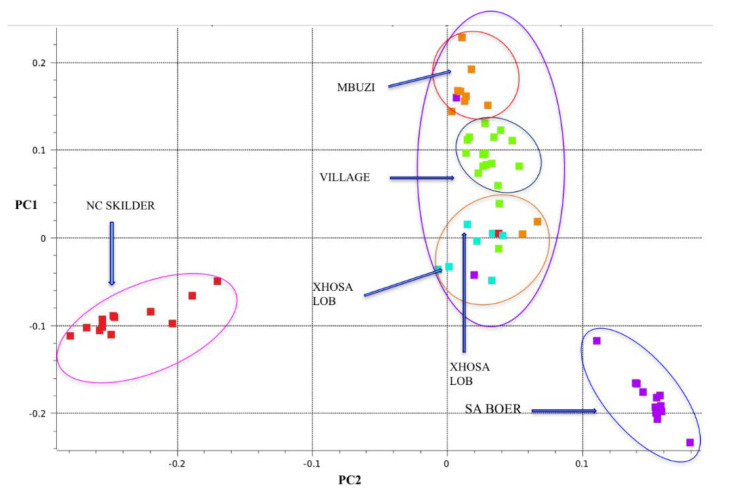
Principal component analysis of the South African indigenous goat populations [13].

The principal component analysis based clustering (Figure 1) produced three main clusters of the (i) Speckled, (ii) Boer goat, and (iii) a cluster that had the Mbuzi, Xhosa, lob eared, and village goats from Pella, as reported in detail in [13]. PC1 separated the NC Speckled from the rest of the populations. PC2 showed variation between the SA Boer and the Village ecotypes. Some of IVG ecotypes clustered with the Tswana goats. The Tswana Village populations formed a cluster in between the XL and the MBZ populations.

### 3.2. Carcass Quality Traits

Means and standard error for carcass quality traits per population are described in Table 1. Breed differences had an effect on all of the traits except for the drip loss percentage (DP), where there were no significant differences between the breeds. The highest pre-slaughter weight (WS) mean was observed in the SAB (40.9 ± 3.6 kg) followed by the NCS (33.1 ± 4.1 kg) goats. The VTE goats had the lowest pre-slaughter weight of 22.0 ± 4.6 kg when compared to the VTI, with an average of 24.2 ± 2.8 kg. The warm carcass weight (WCW) was measured post-slaughter after the animal had been skinned and the head, feet, and offals had been removed.

The SAB and the NCS had the heaviest WCW of 17.2 ± 2.3 kg and 14.1 ± 1.9 kg, respectively. The MBZ, VTE, and the VTI had similar WCW of 9.4 ± 1.5 kg, 9.1 ± 2.0 kg, and 9.9 ± 1.1 kg, respectively. The SAB cold carcass weight (CCW) was the highest, followed by the NCS (16.3 ± 2.3 kg and 9.9 ± 1.1 kg, respectively). The intensively raised village population had a higher CCW than the extensively raised ones (9.2 ± 1.2 kg and 8.4 ± 1.9 kg, respectively).

The SAB, NCS and XL were judged to have a higher fat code (FC) (1.3 ± 0.5, 1.2 ± 0.5 and 1.2 ± 0.4, respectively) compared to MBZ, VTI, and VTE. The VTI had a higher FC (0.4 ± 0.3) than the VTE (0.3 ± 0.0). The NCS produced the highest dressing percentage (DP) (42.5 ± 2.5%), followed by SAB (41.9 ± 2.4%). The chilling loss (CL) was higher in the NCS (8.4 ± 3.5%) population compared to the MBZ and the VTE, which had similar CL proportions (7.4 ± 1.9% and 7.8 ± 2.7%, respectively). The intensively raised VTE had a higher CL (7.8 ± 2.7%) than the VTI population (6.6 ± 2.1%).

The interaction effects of goat breed and sex on the pre-slaughter weight and carcass characteristics are presented in Table 2. The interactions between goat breed and sex were observed for pre-slaughter weight, whereby the XL bucks had heavier carcasses than the does (34.4 kg and 25.8 kg, respectively), while other populations (MBZ, NCS, SAB, VTE, and VTI) had similar live weights for both bucks and does. The warm carcass weight of the XL bucks was higher than that of the does (14.3 kg and 10.5 kg, respectively), while the bucks and does of MBZ, NCS, SAB, VTV, and VT had similar warm carcass weights. The XL bucks had a higher cold carcass weight than that recorded for does (13.3 kg and 9.9 kg), whilst on average, there were no significant differences between the bucks and does of MBZ, NCS, SAB, VTV, and VT in terms of the cold carcass weight. On the other hand, the XL bucks had similar weights compared to NCS (does and bucks combined), and the XL does had similar weights as MBZ, VT, and VTV (does and bucks combined). There were no significant (*p* > 0.05) interaction effects between breeds and sex on the dressing percentage and chilling loss.

### 3.3. Genome-Wide Association

**Figure 2:**
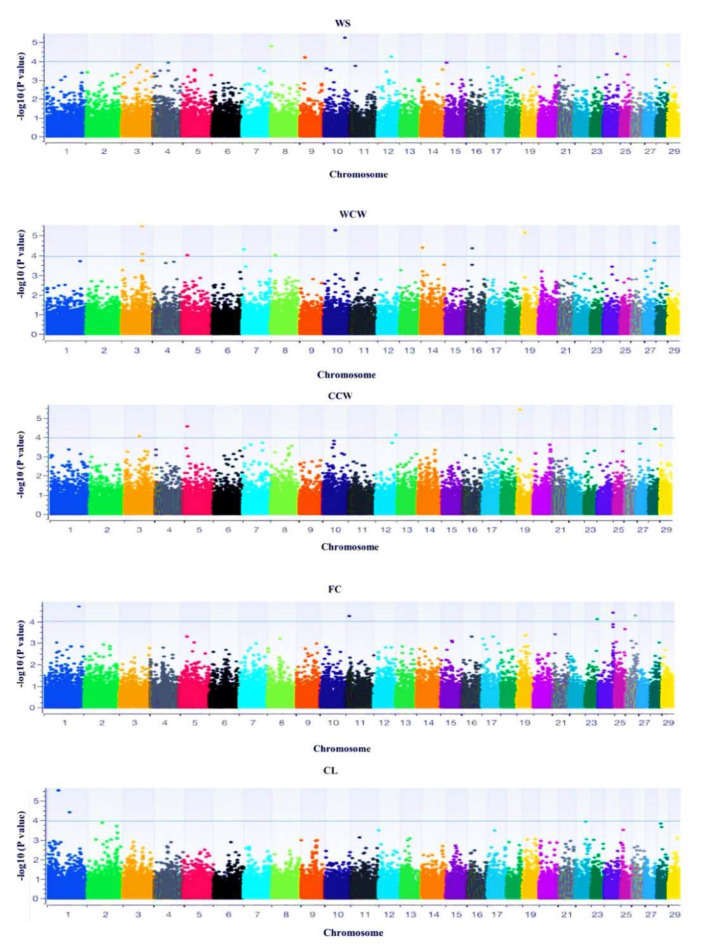
Manhattan plots of the GWAS for carcass quality traits in the South African indigenous goat populations. In the Manhattan plots, Bonferroni adjusted −log10 *p*-values of the quantified SNPs were plotted against their genomic positions; different colours indicate SNPs on different chromosomes from chromosome 1 to 29. WS = slaughter weight, WCW = warm carcass weight, CCW = cold carcass weight, FC = fat code, and CL = chilling loss.

Genome-wide association analysis was undertaken for WS, WCW, CCW, CL, and FC carcass quality traits, and a Bonferroni corrected threshold of (−log10 (*p* < 0.05)) was used to classify the SNPs as being significantly associated, as shown in Figure 2. A total of 40 SNPs were found to be significantly associated (*p* < 0.05) with the above-mentioned carcass traits, and genes were reported within the 1 Mb region of the significant SNPs across different traits (Table 3, Table 4, Table 5 and Table 6). The analysis was performed for the general population as well as individually for the different breeds/populations under study. A total of 40 genomic regions were associated with carcass quality traits and were distributed across 11 chromosomes, as shown in Table 3, Table 4, Table 5 and Table 6. A total of 8 SNPs were significantly associated with pre-slaughter weight, 17 were associated with warm carcass weight, 9 were associated with cold carcass weight, 5 were associated with fat code, and 1 was associated with chilling loss.

Of the eight genes that were significantly associated with WS, the *GADD45G* (growth arrest and DNA damage inducible gamma) was absent in the SAB population, as shown in Table 3, Table 4 and Table 5. Among SNPs associated with WCW, 18 candidate genes were identified in all of the populations. The calpain gene family (*CAPN8* and *CAPN2*) as well as the *VAV3* (Vav guanine nucleotide exchange factor 3) were some of the genes that were associated with WCW. Seven candidate genes were associated with FC, with the *CAPN7* being one of them. *POU1F1* was the only gene that was associated with CL.

## 4. Discussion

Goats are primarily kept and used for chevon production. Local goat populations from South Africa and other developing countries are often characterized as being of low carcass quality and acceptability to consumers [5]. To fill the gap on limited information, this study sought the use five of the South African indigenous populations, including non-descript populations, to investigate carcass characteristics and the associated genetics of South African indigenous goats. The genome-wide association analysis using Illumina goat SNP50K genotypes was used to investigate the genes associated with carcass quality traits, including their molecular functions.

The SAB population had a high pre-slaughter weight compared to the rest of the populations. This was expected, as the Boer has been developed for high meat yield and growth performance [20]. The NCS, which is commonly bred for its hide also showed good growth performance, with a slightly higher slaughter weight than the rest of the Indigenous Veld Goats (33.1 ± 4.1 kg). By comparing the VTI and VTE goats, the study demonstrated the role of management, particularly the role of nutrition, on local breeds. In the extensive system, goats typically browse and fend for their food under harsh climatic conditions [21]; the scarcity of adequate and quality feed contributes to the lower WS in the VTI compared to VTE.

A marketable slaughter weight for goats in their first year is between 30 and 40 kg [12]. The present study showed that only the SAB and NCS fall within that range. However, the variation in the populations that did not meet the market weight are suggestive of potential for selection for optimal growth. 

The slaughter weight corresponded to the carcass yield, and the present study is consistent with that of Pophiwa et al. [20], who reported a higher cold carcass weight (6.1 kg) in Boer goats compared to the indigenous populations (2.9 kg). According to Webb [5], the fat class of carcasses is affected by factors such as sex, body weight, and age, and the breed of the animal. DP is a crucial trait in meat production, as it helps farmers achieve a target live weight before the animals are slaughtered [12]. The DP observed in this study were generally lower than those reported by Tshabalala et al. [22], where a dressing percentage of 55.72 ± 1.58% and 55.68 ± 1.29% was observed in Boer and indigenous goats, respectively. The between breed differences were very small and were in line with Tshabalala et al. [22], who considered goats to be generally lean across breeds. The present study observed high variations of chill loss. Although other studies reported a positive correlation between chilling loss and the live body weight of the animal [20,22], this study reported the SAB to have a higher body weight and lower chill loss (5.5 ± 1.7%), whilst the NCS had a chill loss that was higher (8.4 ± 3.5%) than the rest of the populations. Such a contrasting observation may be due to the effect of breed described by Webb [5], who stated that breed is one of the factors that consequently affects chill loss. 

The study further investigated the SNPs, genes, and pathways associated with carcass quality traits in the SA goat populations using the Illumina goat SNP50K genotype data. The *GADD45G* gene observed for the WS trait control developmental processes, was associated with immune-related functions and plays a role in the regulation of growth and apoptosis, indicating that the extensively raised populations undergo high stress levels [23]. The p53 signaling pathway is involved in the coordination of the stress response [24] and the *GADD45G* gene is one of the genes that plays a role in this pathway, where its enrichment was associated with feed efficiency in beef cattle [23]. In the present study, this gene was not observed in the SAB population, and the reason for the absence is unclear. The village goat production system under harsh climatic conditions contributes to a stressful environment for the non-descript village populations, and the presence of the *GADD45G* gene and the associated P53 signaling pathway attest to that. The *IGF2R* gene, a gene that encodes a transmembrane receptor was associated with WS in the present study. This is consistent with carcass weight and growth traits such as animal body size associations in Irish Holstein-Friesian cattle [25]. With such genes, metabolic and growth-related pathways including the thyroid hormone signaling pathway and immune related pathways such as the cytokine–cytokine receptor interaction pathways were reported, further emphasising the robustness of animals that are able to produce optimally under harsh conditions. Genes that are associated with carcass traits such as WCW, CCW, and fat code as well as with muscle and skeletal development, feed conversion efficiency, and the proteolysis of muscle proteins (i.e., VRTN, VAV3, and calpain gene family members of CAPN8 and CAPN2, respectively) were also found. The VAV3 gene has been found to be associated with meat conversion efficiency in sheep [26] and has also been associated with carcass weight in Hanwoo pigs [27], whilst the calpain genes have been reported to be associated with meat tenderness [28].

Cold carcass weight (CCW) is the weight that is achieved 24 h post-cooling of the carcass and is about 1.5% less compared to the warm carcass weight. The present study is consistent with that of Pophiwa et al. [20], who reported that the Boer goat had higher cold carcass weight (6.1 kg) compared to the indigenous populations (2.9 kg). Eleven candidate genes were significantly associated with this trait and included the growth differentiation factor 11 (*GDF11*), which was reported to have skeletal muscle and bone rejuvenation effects in mice [29]. The transforming growth factor beta receptor 3 (*TGFBR3*) on the other hand, plays an essential role in muscle tissue development in pigs and is associated with pork quality [30] and was associated with CCW in the present study.

Fat code is an important trait, as it affects carcass yield and moisture. According to Webb [5], the fat code of carcasses is affected by factors such as sex, body weight, age, and animal ecotype/breed. In this study, large-framed breeds such as the SAB, NCS, and XL had a high-fat code, while the smaller ecotypes (MBZ, VTI and VTV) had a lower fat content. Seven candidate genes, including the calpain family member (*CAPN7*), were identified. As described above, this gene family and, most specifically *CAPN1,* is associated with meat tenderness. Studies have associated the level of subcutaneous fat with meat tenderness. The tenderness and toughness of meat is therefore dependent on the calpain activity, where high calpain activity post-mortem leads to more tender muscles, and inhibition leads to tougher meat [31]. Mutations in the growth hormone secretagogue receptor (*GHSR*) were associated with the impairment of the constructive activity receptor in human families with short stature [32]. *GHSR* mediates the effects of the Ghrelin hormone, which when administered to pregnant and lactating mice, resulted in foetal and postnatal weight gain [32]. The identification of the *GHSR* gene as a candidate gene for FC implies that this gene plays a role in the growth and development of goats, therefore affecting carcass fatness. This can then be one of the most economically important genes in chevon production. The *POU1F1,* a gene that is associated with chill loss, plays a role in the expression of growth hormone, therefore leading to variations in growth rates. This gene has been associated with weight gain in chickens at various growth stages [33].

## 5. Conclusions

Overall, the study provided some preliminary insights into the carcass quality profiles and genomic regions associated with such carcass traits. The intensively raised goat populations performed better in terms of their weight and carcass characteristics, indicating that improved management can lead to an improvement in growth and carcass traits and the realisation of genetic potential. The observation of the significantly associated SNPs and genes are suggestive of a genetic component that is associated with carcass quality traits, thereby presenting a potential for genetic improvement for chevon production in the South African goat populations. Further studies involving larger sample sizes of the representative breeds are recommended to validate the results of this study and to identify potential genomic markers for use in breed improvement programs. 

## Figures and Tables

**Table 1:** Mean values and standard error for carcass traits of longissimus muscle of indigenous South African goat populations.

	^γ^Breed						
	SAB	NCS	XL	MBZ	VTI	VTE	Significance
* Trait							** (*p < F*) **
WS (kg)	40.9 ^a^ ± 3.6	33.1 ^b^ ± 4.1	29.8 ^c^ ± 5.9	23.0 ^d^ ± 3.4	24.2 ^d^ ± 2.8	22.0 ^d^ ± 4.6	<0.001
WCW (kg)	17.2 ^a^ ± 2.3	14.1 ^b^ ± 1.9	12.2 ^c^ ± 2.6	9.4 ^d^ ± 1.5	9.9 ^d^ ± 1.1	9.1 ^d^ ± 2.0	<0.001
CCW (kg)	16.3 ^a^ ± 2.3	12.9 ^b^ ± 1.7	11.4 ^b^ ± 2.4	8.7 ^c^ ± 1.4	9.2 ^c^ ± 1.2	8.4 ^c^ ± 1.9	<0.001
FC	1.3 ± 0.5	1.1 ± 0.5	1.2 ± 0.4	0.4 ^bc^ ± 0.2	0.4 ^bc^ ± 0.3	0.3 ± 0.00	<0.001
DP (%)	41.9 ± 2.4	42.5 ± 2.5	41.4 ± 1.7	40.9 ± 1.5	40.8 ± 1.7	41.2 ± 2.6	0.353
CL (%)	5.5 ± 1.7	8.4 ± 3.5	6.7 ± 1.7	7.4 ± 1.9	6.6 ± 2.1	7.8 ^y^ ± 2.7	0.059

* Carcass quality traits: WS = weight at slaughter, WCW = warm carcass weight, CCW = cold carcass weight, FC = fat code, CL = chilling loss, and DP (%) = dressing percentage. ^γ^Breed Code: SAB = South African Boer, NCS = Northern Cape Speckled, XLE = Xhosa Lob/Eared, MBZ = Mbuzi, VTI = Village Tswana raised at API research farm, and VTE = Village Tswana raised in extensive conditions at village farms. ^abcd^ Means within a row with different superscripts differ significantly (*p* < 0.05).

**Table 2:** Mean values and standard errors (±SE) for the interaction effects of goat breed and sex on the carcass *m. longissimus dorsi* (LD) characteristics of goat breeds.

	Breed × Sex												Significance *
	SAB		NCS		XL		MBZ		VTI		VTE		
	Does	Bucks	Does	Bucks	Does	Bucks	Does	Bucks	Does	Bucks	Does	Bucks	(*p <* F)
WS (kg)	42.7 ^a^ ± 3.7	39.2 ^ab^ ± 3.0	31.9 ^c^ ± 4.1	34.9 ^bc^ ± 3.7	25.8 ^d^ ± 4.8	34.4 ^c^ ± 2.0	23.9 ^de^ ± 3.4	21.3 ^de^ ± 3.1	24.1 ^de^ ± 3.5	24.4 ^de^ ± 1.9	21.0 ^e^ ± 4.5	23.7 ^de^ ± 5.1	0.004
WCW (kg)	18.0 ^a^ ± 2.5	16.1 ^ab^ ± 1.6	13.4 ^c^ ± 1.9	15.1 ^bc^ ± 1.5	10.5 ^d^ ± 1.9	14.3 ^bc^ ± 1.4	9.7 ^d^ ± 1.5	8.9^d^ ± 1.6	9.9 ^d^ ± 1.4	9.9^d^ ± 0.9	8.5 ^d^ ± 1.7	10.0 ^d^ ± 2.4	0.004
CCW (kg)	17.1 ^a^ ± 2.4	15.0 ^ab^ ± 1.4	12.4 ^c^ ± 1.9	13.6 ^bc^ ± 1.1	9.9 ^d^ ± 1.8	13.3 ^bc^ ± 1.5	9.0 ^d^ ± 1.5	8.2 ^d^ ± 1.4	9.2 ^d^ ± 1.5	9.2^d^ ± 0.9	7.9 ^d^ ± 1.8	9.2 ^d^ ± 2.1	0.006
DP (%)	42.6 ± 2.8	41.0 ± 1.2	42.0 ± 2.4	43.3 ± 2.8	41.3 ± 2.2	41.5 ± 1.1	40.4 ± 0.9	41.7 ± 2.1	40.9 ± 2.3	40.6 ± 1.0	40.6 ± 2.4	42.1 ± 3.1	0.492
CL (%)	4.8 ± 0.8	6.4 ± 2.1	7.5 ± 2.8	9.8 ± 4.2	6.2 ± 1.2	7.3 ± 2.1	7.4 ± 2.3	7.5 ± 1.0	6.5 ± 1.8	6.8 ± 2.8	7.7 ± 3.2	8.0 ± 2.3	0.862

^γ^Breed: SAB = South African Boer, NCS = Northern Cape Speckled, XLE = Xhosa Lob/Eared, MBZ = Mbuzi, VTI = Village Tswana raised at AP research farm, and VTE = Village Tswana raised in extensive conditions at village farms. Means in the same row with different superscripts are significantly different (*p* < 0.05). Carcass quality traits: WS = weight at slaughter, WCW = warm carcass weight, CCW = cold carcass weight, CL = chilling loss percentage, and DP (%) = dressing percentage. * Significance level for breed x sex interaction. ^abcde^ Means within a row with different superscripts differ significantly (*p* < 0.05).

**Table 3:** Marker association with weight at slaughter (WS) in South African goats.

Trait	SNP	Chr	Position	*p*-Value	MAF	Gene	KEGG Pathway
WS	snp10578-scaffold1376-2185653	8	72,415,408	0.005	0.384	*CDCA2*	-
	snp31819-scaffold356-851991	8	99,190,229	0.019	0.289	*MUSK*	-
	snp51334-scaffold750-1385368	8	79,156,981	0.031	0.402	*GAS1*	Hedgehog signaling pathway
	snp42388-scaffold56-1009293	8	87,724,933	0.039	0.100	*GADD45G*	FoxO signaling pathway, p53 signaling pathway, MAPK signaling pathway, NF-kappa B signaling pathway, apoptosis
	snp53226-scaffold802-11917	9	14,759,064	0.002	0.471	*FABP7*	PPAR signaling pathway
	snp36803-scaffold447-444443	9	82,960,991	0.003	0.478	*IGF2R*	Endocytosis, lysosome
	snp39508-scaffold501-3016606	10	17,332,120	0.015	0.435	*VRTN*	-
	snp16896-scaffold1766-13800	25	1,378,654	0.027	0.492	*GFER*	-

**Table 4:** Marker association with warm carcass weight (WCW) in South African goats.

Trait	SNP	Chr	Position	*p*-Value	MAF	Gene	KEGG Pathway
WCW	snp56476-scaffold89-151841	3	11,055,188	0.001	0.42	*THRAP3*	-
	snp44023-scaffold595-5580840	3	89,714,538	0.002	0.196	*CAPZA1*	Endocytosis
	snp5156-scaffold118-634690	3	84,590,504	0.014	0.413	*VAV3*	B cell receptor signaling pathway, Leukocyte transendothelial migration, Rap1 signaling pathway, cAMP signaling pathway, chemokine signaling pathway, focal adhesion, natural kille mediated cyctoxicity, T cell receptor sigbaling pathway, Fc epsilon RI signaling pathway, Yersinia infection, lipid and atheroscierosis
	snp15511-scaffold164-2927845	3	41,839,503	0.039	0.486	*INSL5*	Relaxin signaling pathway, neuroactive ligand receptor interaction
	snp11534-scaffold1421-222892	10	16,932,843	0.001	0.268	*LTBP2*	-
	snp16574-scaffold1748-66549	10	15,612,288	0.005	0.199	*TGFB3*	Cytokine–cytokine receptor interaction, MAPK signaling pathway, FoxO signaling pathway, TGF-beta signaling pathway, Hippo signaling pathway, Chagas disease, leishmaniasis, toxoplamosis, tuberculosis
	snp39508-scaffold501-3016606	10	17,332,120	0.009	0.434	*VRTN*	-
	snp11543-scaffold1421-651926	10	16,503,809	0.002	0.427	*PGF*	MAPK signaling pathway, Ras signaling pathway, Rap1 signaling pathway, focal adhesion, PI3K-Akt signaling pathway, phospholipase D signaling pathway, neuroactive ligand receptor interaction
	snp23543-scaffold237-2500414	14	46,657,313	0.008	0.364	*MSC*	Staphylococcus aureus infection
	snp17242-scaffold18-837754	16	24,869,668	0.012	0.435	*CAPN8*	-
						*CAPN2*	Necroptosis, protein processing in endoplasmic reticulum, apoptosis, cellular senescence, focal adhesion
	snp30146-scaffold331-1127999	16	49,917,608	0.049	0.289	*PERM1*	-
	snp9015-scaffold1328-778798	19	28,743,439	0.012	0.383	*GAS7*	-
	snp43335-scaffold5775-55878	19	55,399,362	0.032	0.478	*GRB2*	Growth hormone synthesis, secretion and action, Insulin signaling pathway, ErbB signaling pathway, T cell receptor sognaling pathway, EGFR tyrosine kinase inhibitor resistance, endocrine resistance, Ras signaling pathway, MAPK signaling pathway, FoxO signaling pathway, mTOR signaling pathway, PI3K-Akt signaling pathway, signaling pathways regulating pluripotency of stem cells, natural killer cell mediated cyctoxity, B cell receptor signaling pathway, Fc epsilon RI signaling pathway, thermogenesis, neurotrophin signaling pathway, JAK-STAT signaling pathway
	snp55723-scaffold864-1874947	27	25,697,142	0.001	0.407	*PDGFRL*	-
	snp30755-scaffold34-2405052	27	4,053,757	0.001	0.478	*THRB*	Thyroid hormone signaling pathway, neuroactive ligand-receptor interaction
	snp44450-scaffold604-977126	27	11,448,725	0.024	0.301	*FGFR1*	Parathyroid hormone synthesis, secretion and action, adherens junction, thermogenesis, MAPK signaling pathway, Ras signaling pathway, Rap1 signaling pathway, calcium signaling pathway, regulation, signaling pathways regulating pluripotency of stem cells
	snp55724-scaffold864-1906315	27	25,665,774	0.039	0.493	*PDGFRL*	-

**Table 5:** Marker association with cold carcass weight (CCW) in South African goats.

Trait	SNP	Chr	Position	*p*-Value	MAF	Gene	KEGG Pathway
CCW	snp11922-scaffold144-3610093	3	68,043,979	0.004	0.435	*TGFBR3*	-
	snp15489-scaffold164-1962025	3	42,805,323	0.005	0.472	*GADD45A*	FoxO signaling pathway, p53 sigaling pathway, MAPK signaling pathway, NF-kapp B signaling pathway, cellular senescence
	snp11896-scaffold144-2409377	3	69,244,695	0.009	0.311	*GFI1*	-
	snp43970-scaffold595-3028771	3	92,266,607	0.020	0.486	*TSHB*	Thyroid hormone synthesis, cAMP signaling pathway Neuroactive-ligand receptor interaction, regulation of lipolysis in adipocytes
						*NGF*	neurotrophin signaling pathway, apoptosis, inflammatory mediator regulation of TRP channesl, aldosterone synthesis and secretion, MAPK signaling pathway, Ras signaling pathway, Rap1 signaling pathway, PI3-Akt signaling pathway, calcium signaling pathway, NF-kappa B signaling pathway, cortisol synthesis and secretion
	snp12164-scaffold1450-542494	5	99,721,587	0.0020	0.478	*DPPA3*	-
						*GDF3*	Cytokine–cytokine receptor interaction
	snp48908-scaffold698-1623875	5	102,631,469	0.023	0.400	*ING4*	Circadian entrainment, retrograde endocannabinoid signaling, relaxin signaling pathway, GABaergic synapse, MAPK signaling pathway, Ras signaling pathway, PI3K-Akt signaling pathway, cardiac muscle contraction, serotonergic synapse, oxytocin signaling pathway
	snp12642-scaffold1482-13523	5	56,734,400	0.029	0.130	*GDF11*	Cytokine–cytokine receptor interaction
	snp43888-scaffold593-832260	5	92,679,764	0.038	0.136	*EPS8*	-
						*RERG*	-
	snp10950-scaffold1393-138987	5	30,292,487	0.042	0.261	*LMBR1L*	-

**Table 6:** Marker association with fat classification (FC) in South African goats.

Trait	SNP	Chr	Position	*p*-Value	MAF	Gene	KEGG Pathway
FC	snp26446-scaffold276-6029619	1	94,587,913	0.011	0.420	*GHSR*	Growth hormone synthesis, secretion, and action; neuroactive ligand receptor interaction, cAMP signaling pathway
	snp37534-scaffold46-1530591	1	151,789,185	0.028	0.272	*COL6A5*	ECM–receptor interaction, PI3K-Akt signaling pathway, focal adhesion, protein digestion and absorption
						*COLQ*	-
						*CAPN7*	-
	snp36302-scaffold435-1967666	1	74,307,126	0.029	0.351	*FGF12*	-
	snp23054-scaffold230-2979377	1	130,211,629	0.032	0.100	*MRAS*	Ras signaling pathway, C-type lectin receptor signaling pathway, MAPK signaling pathway, Rap1 signaling pathway, phospholipase D signaling pathway, cellular senescence, apelin signaling pathway, regulation of cytoskeleton
	snp20787-scaffold204-4829802	11	77,889,065	0.042	0.447	*LDAH*	-
						*GDF7*	Axon guidance, cytokine–cytokine receptor interaction, TGF-beta signaling pathway, Hippo signaling pathway

## Data Availability

The datasets that were analysed during the current study are available from the corresponding author upon reasonable request.
